# Clinical and Radiological Outcomes of Extreme Lateral Interbody Fusion (XLIF) in the Treatment of Lumbar Spondylodiscitis: A Multi-center Study

**DOI:** 10.7759/cureus.76356

**Published:** 2024-12-25

**Authors:** Yixiang Tan, Fadzrul Abbas Mohamed Ramlee, Mohd Hezery Harun, Mohd Shahril Jaapar, Chor Ngee Tan

**Affiliations:** 1 Orthopedics, Hospital Sultan Abdul Aziz Shah, Serdang, MYS; 2 Orthopedics, Hospital Putrajaya, Putrajaya, MYS; 3 Orthopedic Surgery, Universiti Putra Malaysia, Serdang, MYS; 4 Orthopedics and Traumatology, Universiti Putra Malaysia, Serdang, MYS

**Keywords:** clinical outcome, lumbar, radiological outcome, spondylodiscitis, xlif

## Abstract

Introduction

Lumbar pyogenic spondylodiscitis is a challenging and rare spinal infection with high morbidity, particularly in patients with comorbidities. While the extreme lateral interbody fusion (XLIF) technique is established in treating degenerative spinal conditions, its efficacy in managing spondylodiscitis is less well-studied. This study aims to evaluate the clinical and radiographic outcomes of the XLIF approach combined with posterior instrumentation in patients with lumbar spondylodiscitis.

Method

In a multi-center, retrospective cohort study, 15 patients (mean age 66; 62.5% male) diagnosed with pyogenic spondylodiscitis underwent XLIF with posterior percutaneous fixation between January 2018 and December 2022 at two Malaysian hospitals. Patients were selected based on strict inclusion criteria, including a confirmed single-level disc infection and a minimum follow-up of one year. Clinical outcomes, including Visual Analog Scale (VAS) pain scores, infection markers (C-reactive protein (CRP), erythrocyte sedimentation rate (ESR), white blood cell (WBC)), and fusion rates, were recorded preoperatively and at six weeks, three months, six months, and one year postoperatively. Data were analyzed using appropriate statistical tests, with a significance level set at p<0.05.

Results

The study demonstrated a significant reduction in VAS scores, from a mean of 7 preoperatively to 1.87 at six weeks postoperatively, representing a 73.3% pain reduction (p<0.005). Mean blood loss was minimal (193.3 mL), and no patients required transfusions. Marked reductions in inflammatory markers were observed, with CRP, ESR, and WBC levels decreasing by 75%, 71.5%, and 38.5%, respectively, within the first six weeks (p<0.005). Radiological assessment showed a 100% fusion rate with a mean fusion time of 4.1 months. Complications were low, with only one case of screw malposition and one surgical site infection, both managed without further morbidity.

Conclusion

The XLIF approach with posterior instrumentation is a safe and effective intervention for lumbar spondylodiscitis, providing substantial pain relief, infection control, and reliable spinal fusion. These findings suggest XLIF as a viable surgical option for lumbar spondylodiscitis, especially for patients with multiple comorbidities, warranting consideration as a primary surgical strategy for this challenging condition.

## Introduction

Lumbar pyogenic spondylodiscitis, a rare disorder, accounts for 2-7% of musculoskeletal osteomyelitis cases. This condition persists with a high incidence in developing nations, accompanied by a significantly elevated mortality rate [1]. Low back pain, fever, severe radicular thigh or leg pain, and neurological deterioration are the primary clinical manifestations of lumbar pyogenic spondylodiscitis. However, there remains controversy regarding the optimal surgical approach and instrumentation modality for management [1]. Surgical intervention should be considered in cases where there is neurological dysfunction, significant structural compromise of the vertebrae, the presence of an epidural abscess, unsuccessful conservative therapy, or severe and persistent back pain that does not respond to non-surgical treatments.

Patients who underwent surgical stabilization experienced improved clinical outcomes, including faster recovery, decreased pain levels, and enhanced quality of life, compared to those managed with bracing and antibiotic therapy at one, three, and six months following the intervention [2]. High rates of infection clearance and fusion have been achieved through trans-abdominal approaches, though these methods necessitate extensive surgical exposures and carry non-negligible risks of wound, vascular, and peritoneal complications [3].

The lateral trans-psoas interbody fusion technique, particularly the extreme lateral interbody fusion (XLIF) approach, is a minimally invasive, lateral, retroperitoneal surgical procedure targeting the anterior spinal column. This method involves the manual dissection of the retroperitoneal space, which reduces injury to the surrounding muscles and adjacent structures. Access to the psoas muscle is obtained through its attachment to the transverse processes of lumbar vertebrae. Intraoperative neurophysiology monitoring is utilized when passing through the psoas muscle, and the retraction system is expanded with direct observation of the surgical field. A large interbody cage is then placed to maximize intervertebral space. The XLIF technique is considered an indirect decompression surgery, as its primary goal is to restore disc and foraminal height, leading to symptomatic relief, which represents its key advantage over more invasive decompression and interbody fusion procedures [4]. This method preserves the anterior longitudinal ligament and provides adequate visualization of the intervertebral discs, enabling thorough debridement and the placement of a sizeable, lordotic cage [5].

The clinical benefits of the XLIF approach have been well-documented in the management of degenerative spinal conditions. However, its effectiveness in the treatment of spondylodiscitis has not been extensively studied [3]. This surgical technique allows for the separation of the infected anterior spinal column, which can be thoroughly debrided, from the relatively unaffected posterior column. This enables the application of percutaneous pedicle screw fixation, which maximizes the stability required for successful spinal fusion and may potentially reduce the incidence of cage subsidence. While other all-posterior approaches, such as transforaminal lumbar interbody fusion (TLIF) with pedicle screws can be done, they may risk contaminating the posterior column and increasing the potential for implant infection, which could ultimately lead to implant failure [1].

Although our centers have reported favorable outcomes with this approach, the existing literature on this topic remains limited, with only approximately 10 published articles and no local studies conducted here in Malaysia. To contribute to this field, we aim to investigate the outcomes of this treatment approach in a more rigorous and scientific manner. Our goal is to provide evidence that supports the utilization of this approach in the management of lumbar spondylodiscitis.

## Materials and methods

Patient selection

This study included a total of 15 patients who underwent surgery using the XLIF from January 1, 2018, to December 31, 2022, in Hospital Putrajaya and Hospital Sultan Abdul Aziz Shah. Institutional review board approval was obtained from the Medical Research and Ethics Committee (MREC) of the Ministry of Health (MOH) Malaysia (approval number: 23-02526-G0D). The inclusion criteria for the study required that patients have a primary diagnosis of pyogenic spondylodiscitis due to hematogenous spread, involving a single level of discitis. Diagnosis had to be confirmed through radiological, biochemical, and clinical findings, with a minimum follow-up period of one year. Patients were excluded if they had previous spine surgery or implant infections, fungal infections, or tuberculosis or if their follow-up was less than one year. All patients underwent the XLIF procedure which includes a lateral trans-psoas approach for debridement, endplate preparation, and interbody fusion using a polyetheretherketone (PEEK) cage, followed by percutaneous posterior instrumentation for stabilization. All patients were treated with broad-spectrum empirical antibiotics if the culture was negative or specific to the culture results until the normalization of infective markers.

Research method

We performed a retrospective cohort study during the specified time frame. All surgical procedures were carried out using consistent techniques and methods by experienced spine surgeons. Intraoperative details were documented, including operative duration, estimated blood loss, fusion levels, and culture and sensitivity findings. Clinical outcomes were evaluated using the Visual Analog Scale (VAS) for back and leg pain, infective biomarkers, and length of hospital stay. Radiographic assessment encompassed the evaluation of bony fusion and potential complications such as cage subsidence or implant failure. Clinical outcomes and imaging studies, including anteroposterior and lateral X-rays, were assessed preoperatively and at six weeks, three months, six months, and one year postoperatively. The assessment of fusion was based on a combination of radiological and clinical evidence. The radiological assessment involved examining plain radiographs for evidence of anterior bone bridge and the absence of radiolucency around the cage. Clinically, patients were evaluated to ensure there was no back pain indicative of instability. In the case of instability pain, the patient will be further investigated for non-fusion by proceeding with a computed tomography (CT) scan. It is acknowledged that plain radiographs may overestimate the fusion rates compared to more detailed CT scans. Nonetheless, standard radiographs effectively demonstrate the positioning and integrity of the implants, as well as the presence of any deformities. For patients who do not achieve the desired clinical outcomes, a more comprehensive evaluation of spinal fusion using advanced imaging techniques may be necessary [6]. 

Descriptive statistical analyses were conducted for selected variables. The findings were presented based on the types and distribution of the data. Categorical data were summarized as frequencies and percentages, while numerical data were reported as means and standard deviations, or as medians and interquartile ranges, as appropriate. Comparisons of the differences between two sets of normally distributed numerical data within the same group were analyzed using the paired t-test, Friedman test, or ANOVA test. Alternatively, the Wilcoxon signed-rank test was employed if the data were not normally distributed. All probability values were two-sided, and a p-value of less than 0.05 was considered statistically significant.

## Results

A total of 15 patients underwent the XLIF procedure during the study period. The age range spans from 49 to 82 years, with most patients (37.5%) falling within the 61-70 age group. A majority (62.5%) of patients were male. The main indication for operation includes severe back pain in six cases (40%) and neurological symptoms in five cases (33.3%). The other indications were a combination of both (two cases) and epidural abscess (two cases). The most common infection level is L2/L3, accounting for 37.5% of cases. Diabetes mellitus (60%) was the most prevalent comorbidity among patients. The demographic data is summarized in Table 1.

**Table 1:** Demographic data

Category	Characteristic	Value	Percentage/additional info
Age	Mean	66	Years
	Range	49-82	Years
	Age groups		<50: 1 (6.2%)
			50-60: 3 (18.8%)
			61-70: 6 (37.5%)
			71-80: 3 (18.8%)
			>80: 2 (12.5%)
Gender	Male	10	62.5%
	Female	5	31.2%
Level of infection	T12/L1	2	12.5%
	L2/L3	6	37.5%
	L3/L4	2	12.5%
	L4/L5	4	25%
Hospital stay (days)	24.87±13.63		
Estimated blood loss (mL)	193.33±125.2		
Operative time (minutes)	196.3±39		
Complications	2 (15)	Screw malposition	1 (6.6%)
		Surgical site infection	1 (6.6%)

**Figure 1:**
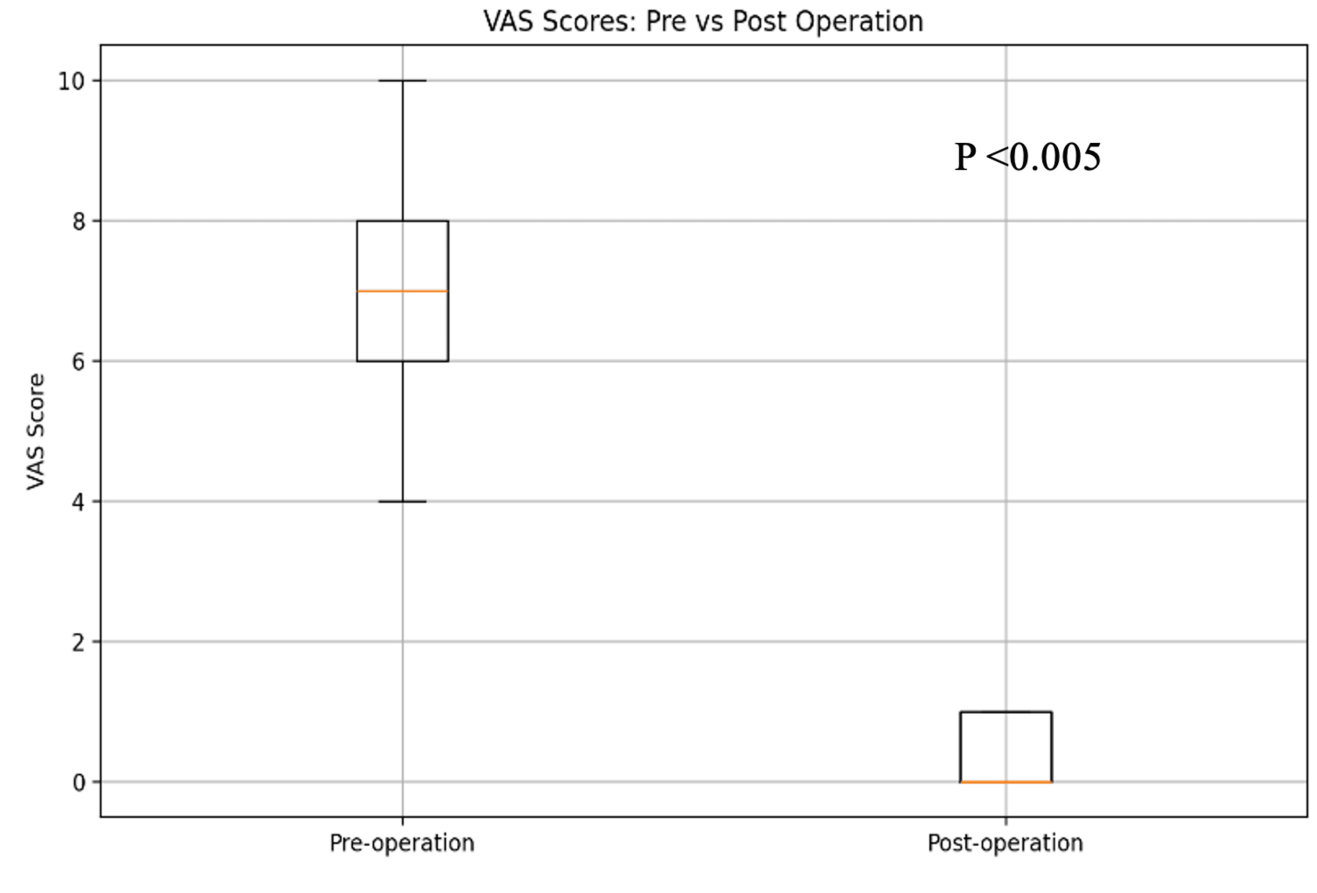
Mean VAS score comparison between pre- and post-operation VAS: Visual Analog Scale

**Figure 2:**
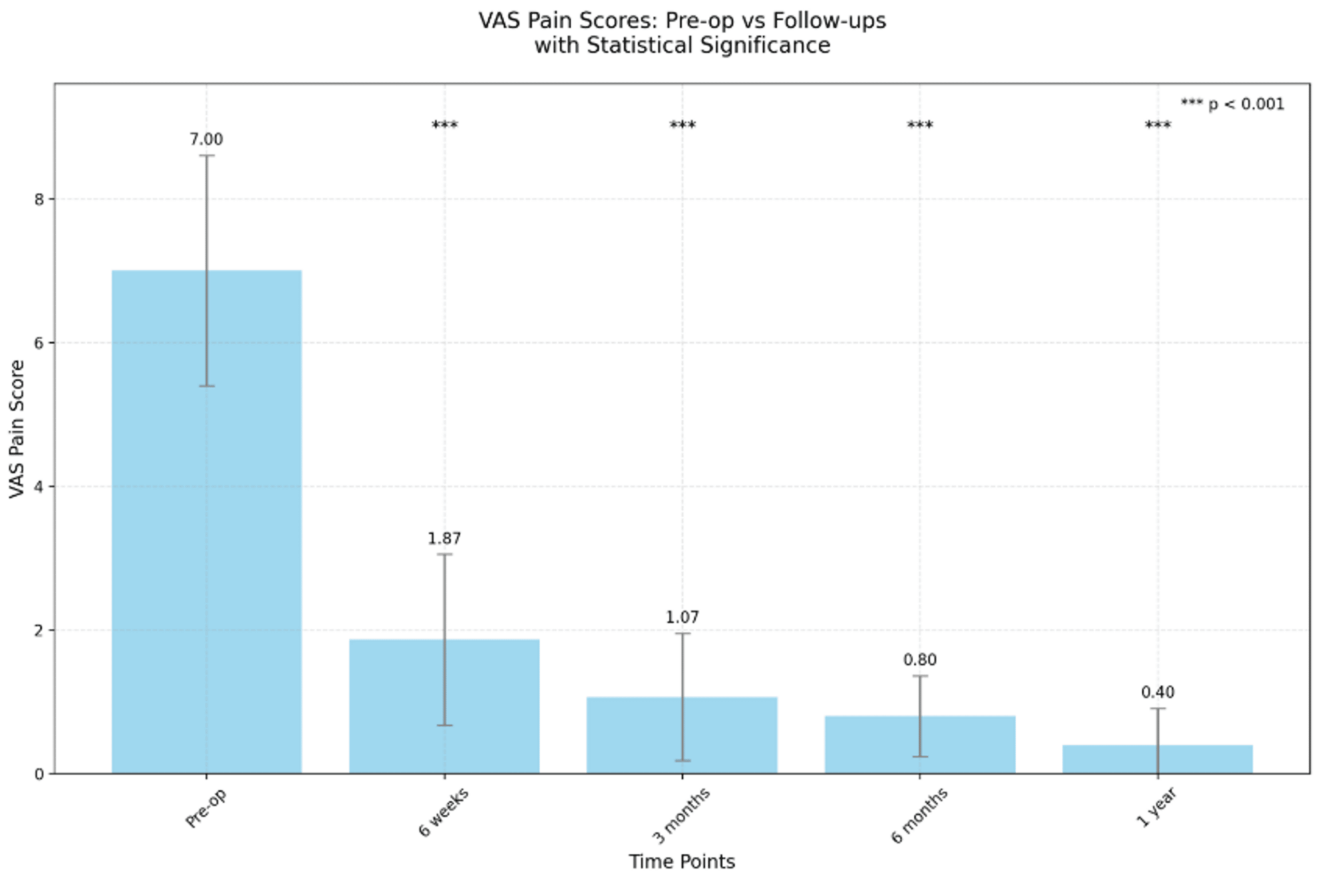
Mean VAS score across different time points during follow-up VAS: Visual Analog Scale

The analysis of the VAS pain scores demonstrated a statistical significance of diminishing pain postoperatively (Figure 1). The mean preoperative VAS was 7.0±1.6, while the mean VAS at six weeks postoperatively decreased substantially to 1.87±1.1. The Friedman test yielded a statistically significant p-value of <0.005 postoperatively during each follow-up, indicating that the pain scores differed significantly across the various time points (Figure 2). The greatest reduction in VAS was observed within the initial six weeks following the surgical procedure which was 73.3%. 

**Figure 3:**
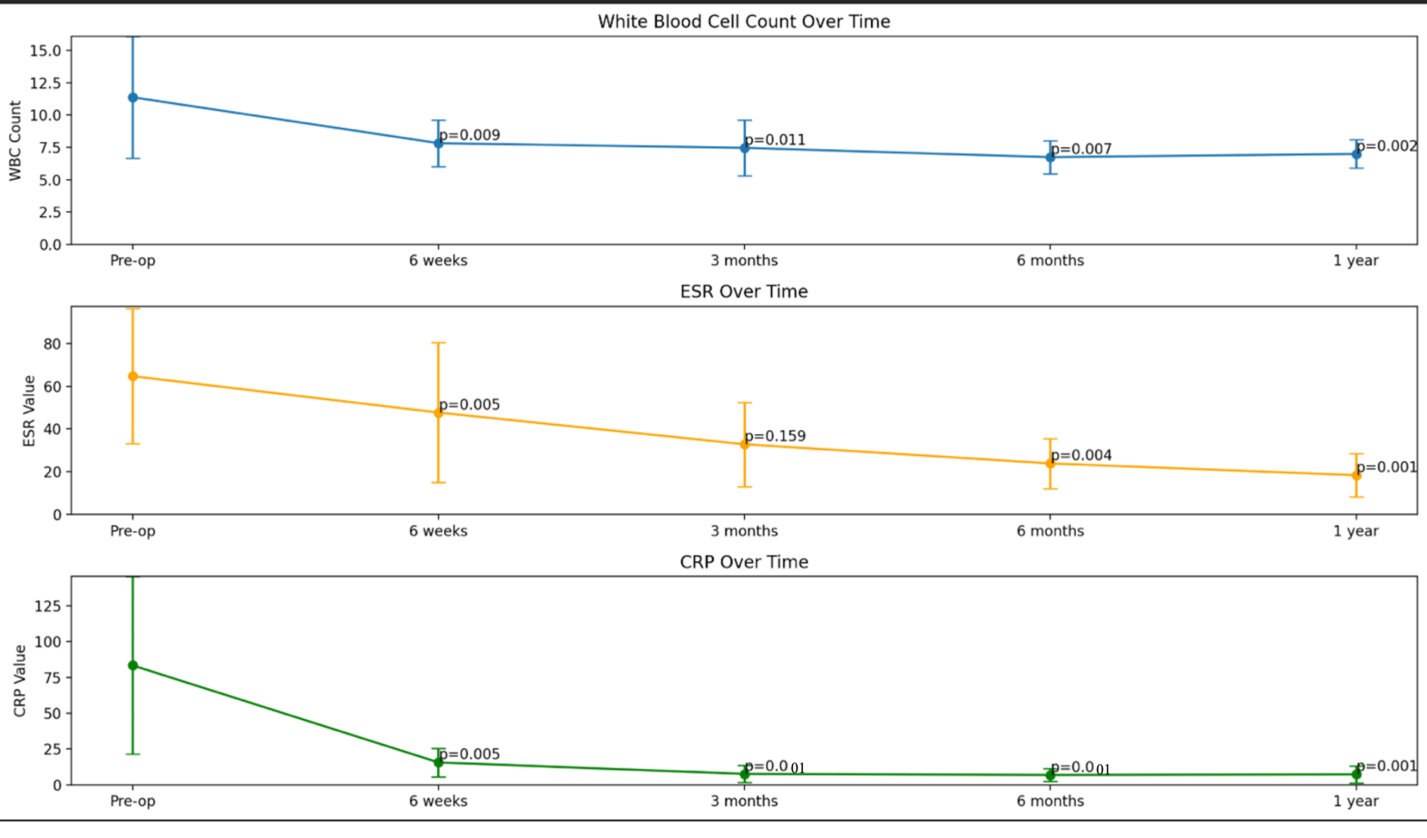
Mean level of infective markers (WBC count, ESR, and CRP) at different time of follow-up All three graphs showed reduction of infective markers over time and normalization at six weeks postoperatively WBC: white blood cell; ESR: erythrocyte sedimentation rate; CRP: C-reactive protein

The mean estimated blood loss was moderate at 193.33±125.2 mL. None of the patients required blood transfusion intraoperatively. The mean operative duration was approximately 196.3±39 minutes, which included the time necessary for comprehensive debridement and patient positioning across the lateral and prone positions. There were substantial reductions in inflammatory markers over time, with C-reactive protein (CRP) decreasing by 75%, erythrocyte sedimentation rate (ESR) by 71.5%, and white blood cell (WBC) by 38.5% within the first six weeks of operation. The difference across all time points was statistically significant (p<0.005) (Figure 3). The majority (73.3%) of patients achieved normalized CRP levels within three months after the surgical intervention. Culture and sensitivity results from the disc material debrided were negative in most cases, with only three returning positive results, which included *Staphylococcus aureus*, *Achromobacter *sp., and *Streptococcus viridans*. The majority of patients (10 out of 15) regained ambulatory status within 30 days, while the others had a longer recovery period for ambulation due to the presence of neurology preoperatively.

**Figure 4:**
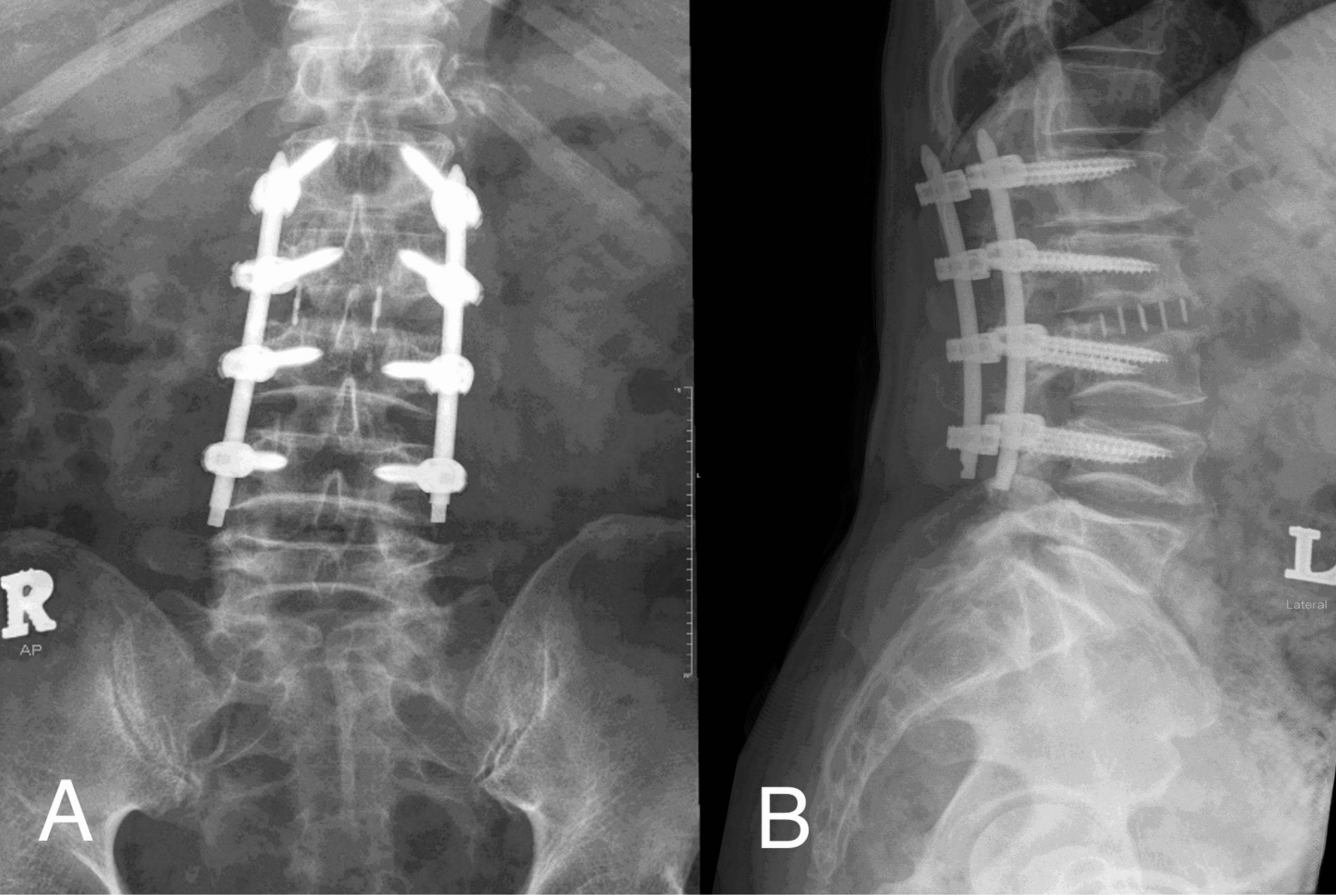
Postoperative lumbo-sacral X-ray to evaluate the placement of implant and fusion (A) Antero-posterior view showing the cage placement which included bi-cortical extension of the vertebral body. The alignment was restored and added further stability to the construct (B) Lateral view to evaluate the restoration of sagittal balance and the assessment of fusion at the level of cage placement

The radiological outcomes demonstrated a 100% fusion rate across all cases. An example of a postoperative radiograph is shown in Figure 4. The majority of patients achieved this solid bony fusion within 3-4 months with a mean time of 4.1 months following the surgical intervention. Only one patient experienced cage subsidence, but fusion was ultimately achieved. Only two patients experienced postoperative complications (1× screw malposition and 1× surgical site infection). Both complications were effectively managed with no further morbidity. 

## Discussion

The study population in this cohort reflected the demographic profile of individuals affected by lumbar spine infections, with a mean age of 66 years and a male predominance of 62.5%. Lumbar spondylodiscitis tends to afflict middle-aged to elderly patients, as well as a higher preponderance in men, consistent with the findings [7]. The study population demonstrated a high prevalence of diabetes mellitus (60%) as the associated comorbidity. Diabetes mellitus is a significant predisposing factor for spinal infections due to its negative impact on immune function and vascular health, which aligns with the increased risk of spondylodiscitis in diabetic populations [8]. However, the successful outcomes observed in this study, despite the patients' high prevalence of comorbid conditions, emphasize the procedure's versatility and ability to manage high-risk patient populations more effectively than open surgical techniques such as the anterior approach. 

Our data reveal a marked reduction in pain scores, as indicated by the VAS, with mean scores dropping from 7 preoperatively to 0.4 at one-year follow-up: a 94.3% reduction. The significance of this reduction, confirmed through statistical testing (Figure 1), reflects the procedure's robust efficacy in pain management, with most pain alleviation occurring within the first six weeks of surgery. Timothy et al. performed a study on 14 patients with lumbar spondylodiscitis undergoing XLIF, reporting similar outcomes with statistically significant improvements in VAS at early follow-up [3]. Blizzard et al. published a study involving 11 patients, all of whom were treated with the XLIF procedure and subsequent posterior fixation. The authors reported that all patients in their cohort achieved symptom resolution and did not require additional debridement [5]. Our result is consistent with current literature on minimally invasive spinal procedures, which often demonstrate rapid symptom relief and functional gains [4]. This corroborates the utility of the XLIF approach in effectively treating pain associated with spondylodiscitis.

The procedure's safety profile is reinforced by minimal estimated blood loss (mean of 193.3 mL) and mean operative duration of 196 minutes, and this amount of blood loss and duration of surgery is comparable or superior to previous studies published using a similar approach [9,10]. In contrast, our data also demonstrated the superiority of the lateral approach in terms of blood loss compared to conventional anterior surgical techniques. For instance, Pee et al. reported a mean blood loss of 810±419 mL in patients treated with anterior debridement and fusion followed by posterior pedicle screw fixation [11]. The low complication rate, with only two cases, namely, screw malposition (revised) and surgical site infection (treated conservatively), demonstrates a high degree of procedural safety, especially pertinent in a patient cohort characterized by advanced age and comorbidities.

The radiographic outcomes were excellent with a 100% success rate among study participants. The fusion time analysis shows a mean fusion time of approximately 4.1 months, with a moderate positive correlation (0.47) between fusion time and age, suggesting older patients may experience longer fusion times though all remained within clinically acceptable limits. The results of this study demonstrate the reliability of XLIF in successful spinal fusion, supporting its consideration as a primary surgical strategy for spinal infections requiring stabilization. This is consistent with the current literature, which have also reported a 100% fusion rate in patients treated with the lateral trans-psoas interbody fusion technique [3]. This high rate of fusion aligns with findings from other studies that utilized the same surgical approach [9,3]. The uniformity in fusion outcomes across these studies reinforces the evidence base for the lateral approach in achieving stable, long-lasting results in diverse patient populations.

Substantial decreases in inflammatory biomarkers were observed throughout the follow-up period, indicating the procedure's efficacy in addressing the underlying infectious process. This provides additional evidence of successful infection control. Specifically, CRP levels decreased by 75%, ESR by 71.5%, and WBC by 38.5% over the follow-up period, all of which were statistically significant as shown in Figure 2. Blizzard et al. observed a complete resolution of infective biomarkers in their patient cohort within one year of follow-up [5]. These findings suggest XLIF is effective in managing lumbar spondylodiscitis, allowing for both rapid symptom relief and long-term infection control. As for the culture and sensitivity results, only three out of 15 patients returned as positive and all were from blood culture. For culture-negative spondylodiscitis, they were all treated with empirical broad-spectrum antibiotics until the normalization of infective markers.

Overall, the results of this study prove the clinical and radiological effectiveness of the lateral debridement and posterior instrumentation approach in the treatment of lumbar spondylodiscitis. Compared to traditional open surgical approaches, XLIF utilized a less invasive method of accessing the infected disc space while preserving the posterior spine column and minimizing soft tissue trauma. This approach confers several key advantages, including shorter operative times, reduced blood loss, and quicker patient mobilization, ultimately leading to shorter hospital stays and faster recovery [12,13]. Additionally, this technique has been shown to provide sufficient access for thorough debridement of infected disc spaces while also allowing for the placement of relatively larger structural interbody cages to restore disc height and facilitate bony fusion [12-14]. The findings of this study are consistent with the existing literature, which has demonstrated the clinical efficacy and radiological benefits of the lateral approach in the management of various spinal pathologies [12,13]. 

Limitations

This study has several limitations that should be acknowledged. The small sample size of 15 patients limits the generalizability of the findings, and the geographic restriction to two hospitals in Malaysia may not reflect the general population. Furthermore, the retrospective nature of the study may lead to potential biases, as it relies on existing records rather than prospective data collection. Additionally, the follow-up period of at least one year may overlook longer-term complications such as junctional syndrome. The reliance on plain radiographs for assessing fusion rates, rather than advanced imaging techniques like CT scans, may lead to overestimation. Moreover, the absence of a control or comparison group, such as patients treated with alternative techniques, and the lack of randomization weaken the ability to draw conclusions about the superiority of the XLIF procedure.

Further limitations include the empirical treatment of culture-negative infections, which hinders the evaluation of specific antibiotic regimens. Strict inclusion criteria might have excluded patients with more complex conditions, introducing selection bias. The study's outcomes, achieved by experienced surgeons, may not be replicable in less specialized settings, and the findings do not encompass younger or pediatric populations. Finally, the study emphasizes clinical and radiological metrics but lacks comprehensive data on functional recovery, quality of life, or return to work, limiting its scope in evaluating overall functional outcomes.

## Conclusions

This study confirms the efficacy of XLIF as a safe, effective intervention for lumbar spondylodiscitis. The procedure's high success rates in achieving pain relief, clinical improvement, and fusion highlight its clinical value. The promising outcomes presented here warrant further investigation into its broader application and underscore its potential to enhance the quality of life in patients contending with spinal infections, particularly those with multiple comorbidities. The findings also align closely with the study's objectives, offering critical insights that may inform future treatment guidelines as the gold standard treatment for lumbar spondylodiscitis.
